# Experimental and Theoretical Studies in Hydrogen-Bonding Organocatalysis

**DOI:** 10.3390/molecules200915500

**Published:** 2015-08-26

**Authors:** Matej Žabka, Radovan Šebesta

**Affiliations:** Department of Organic Chemistry, Faculty of Natural Sciences, Comenius University in Bratislava, Mlynská dolina, Ilkovičova 6, Bratislava SK-842 15, Slovakia; E-Mail: zabka.matej@gmail.com

**Keywords:** organocatalysis, hydrogen bonding, thiourea, squaramide, experimental and computational studies, reaction mechanisms

## Abstract

Chiral thioureas and squaramides are among the most prominent hydrogen-bond bifunctional organocatalysts now extensively used for various transformations, including aldol, Michael, Mannich and Diels-Alder reactions. More importantly, the experimental and computational study of the mode of activation has begun to attract considerable attention. Various experimental, spectroscopic and calculation methods are now frequently used, often as an integrated approach, to establish the reaction mechanism, the mode of activation or explain the stereochemical outcome of the reaction. This article comprises several case studies, sorted according to the method used in their study. The aim of this review is to give the investigators an overview of the methods currently utilized for mechanistic investigations in hydrogen-bonding organocatalysis.

## 1. Introduction

Organocatalysis using chiral hydrogen-bond (H-bond) donor catalysts, such as thioureas and squaramides, has expanded rapidly in the past few years. This type of catalysis now efficiently addresses the issue of the formation of multiple chiral centers with high stereoselectivity [1,2,3,4,5,6,7,8,9]. Thus, the rapid construction of complex molecules from simple starting material is enabled, often with high functional group tolerance. Only recently, many research groups began to offer insights into the reaction mechanisms and the modes of action of the H-bond catalysts, which include complex formation with cations, anions, and neutral molecules [10,11]. Both experimental, such as spectroscopic (NMR, IR, kinetics, *etc.*) and computational studies have been called upon to gain deeper understanding of the way how these catalysts function. This information could prove advantageous in choosing a more suitable catalyst for the particular transformation or designing catalysts for new reactions. IUPAC defined H-bond as “an attractive interaction between a hydrogen atom from a molecule or a molecular fragment X–H in which X is more electronegative than H, and an atom or a group of atoms in the same or a different molecule, in which there is evidence of bond formation” [12]. As no covalent intermediates of the substrate with the catalyst are formed, in contrast to enamine/iminium organocatalysis [13,14,15,16,17,18], the isolation of reactive intermediates is often not possible. Moreover, a broad range of hydrogen-bond strengths (H-bonds are usually considerably weaker than typical covalent bonds, encompassing interactions in the range of 0.8–167 kJ·mol^−1^) and small association constants render the intermediates difficult to be studied directly by spectroscopy [19]. Although there are also other types of H-bonding organocatalysts [8,20,21], this article focuses on the two most important classes of such catalysts, thioureas and squaramides.

## 2. Experimental Techniques

Although mechanistic studies in covalent organocatalysis are not necessarily easier, the covalent bond between substrates and catalysts makes it inherently more amenable to investigation [17,22]. On the other hand, weaker interactions in hydrogen-bonding catalysis make it more difficult to study. However, there has been numerous attempts to understand mechanisms of hydrogen bonding catalysis. The majority of our knowledge stems from spectroscopic, kinetic and computational investigations, or their combinations.

### 2.1. Determination of Catalyst Acidity: UV-Spectrophotometric Methods

**Figure 1:**
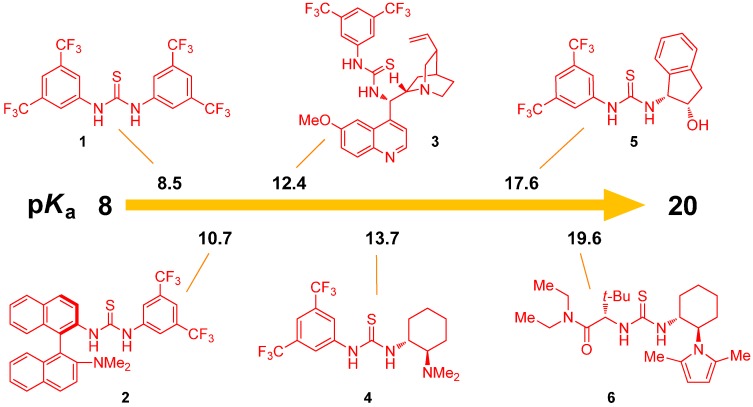
Equilibrium acidities of various thioureas in DMSO.

The equilibrium acidities are the most straightforward data that could be utilized to rank H-bond catalysts, according to the strength of hydrogen bond. Although these catalysts are supposed to act as H-bond donors and not Brønsted acids, this scale should nevertheless represent a sufficient/rough model for activity quantification. Rueping, Gschwind, and coworkers used NMR to distinguish between H-bonding and ion-pairing in Brønsted acid catalysis [23]. Equilibrium acidities of the most popular (thio)urea organocatalysts were measured in dimethylsulfoxide (DMSO) using the UV-spectrophotometric method of overlapping indicators, developed by Bordwell [24]. Catalysts have p*K*_a_ values in the relatively broad range of 8.5–20 (Figure 1), thus providing a scale of their capability to act as H-bond donors. The replacement of urea with thiourea moiety, the addition of aryl substituents and the introduction of CF_3_ groups to the aryl rings attached to the thiourea moiety all led to an increase in acidity. Thus, thiourea **2** represents the most acidic chiral catalyst, as the other catalysts possess at least one aliphatic substituent.

Turnover frequencies correlate with the catalyst acidities for a selection of model reactions from the literature. The higher acidity of the catalyst also resulted in its higher catalytic activity. However, the authors concluded that the acid strength is only one of the parameters defining the catalytic activity.

**Figure 2:**
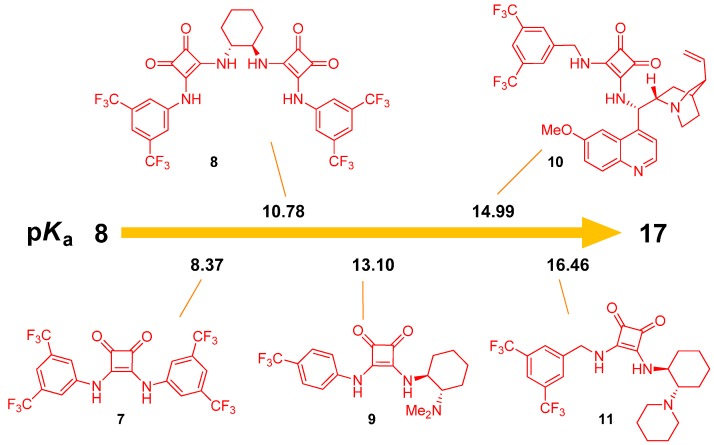
Equilibrium acidities of various squaramides in DMSO.

Squaramides offer better catalytic activities than the corresponding thioureas. The possible reasons behind this include the coplanarity of two N–H bonds with the cyclobutenedione, a larger distance between these N–H bonds (by 0.6 A) and the difference in dihedral angle by 8° [25]. The equilibrium acidities of various squaramides were also determined using the same method of overlapping indicators (Figure 2) [26]. Similarly to thioureas, the introduction of electron-withdrawing CF_3_ groups into the squaramide structure increases its acidity. There seems to be a correlation between the p*K*_a_ values of squaramides and thioureas of analogous structure. Squaramides typically have p*K*_a_’s lower by 0.13–1.97 units than the corresponding thioureas, which presumably leads to stronger H-bond and increased catalytic activity. The overall range for squaramides is 8.3–16.5 p*K*_a_ units.

**Figure 3:**
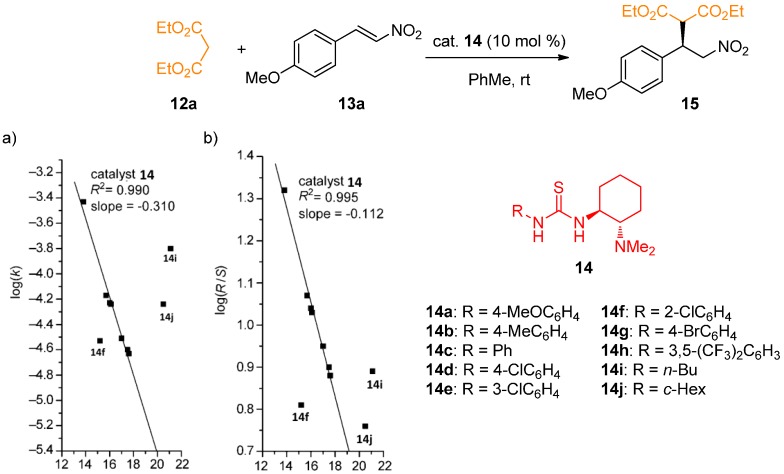
Correlations of p*K*_a_ values with (**a**) reaction rates; (**b**) enantioselectivities for the Michael addition of diethyl malonate to nitrostyrene **13a**. Adapted with permission from Ref. [27]. Copyright (2009) John Wiley and Sons.

Luo, Cheng, and coworkers studied the linear free-energy relationship of thiourea acidity with the catalytic activity and stereoselectivity on the Michael addition of diethyl malonate (**12a**) to nitrostyrene **13a** and related Michael reactions [27]. After screening many catalysts, a linear correlation was observed. Negative slopes reflect the overall trend in all the correlations: More acidic catalyst in a structurally related series leads to the faster reaction and increased enantioselectivity. However, some deviations were observed for *ortho*-substituted aromatic thioureas. The example shows the correlations observed with Takemoto-type thioureas (Figure 3).

**Figure 4:**
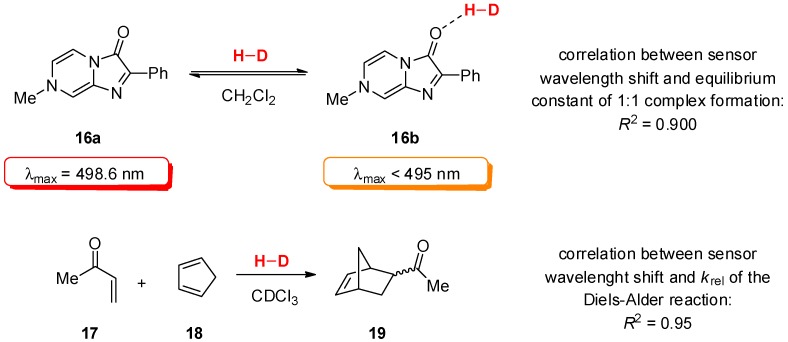
UV-Vis sensor and the correlation between sensor wavelength shift and sensor-catalyst binding equilibrium constant.

However, the analysis of p*K*_a_ values do not take into account various secondary interactions, mainly steric effects, and binding geometry. A different approach to assessment of H-bond catalysts relies on a spectrophotometric sensor **16a** and its ability to respond to LUMO-lowering imparted by hydrogen bonding of the carbonyl group by shifting its λ_max_ [28]. This change in electronic properties of the sensor is, therefore, detectable by UV-Vis spectroscopy (Figure 4). A plethora of H-bond donors, including phenols, carboxylic acids, ammonium salts, (thio)ureas and squaramides were evaluated. A 1:1 binding stoichiometry with the sensor molecule and rapid equilibration were revealed. The relevance of the obtained data was exemplified by the kinetic studies of Diels-Alder reaction between methyl vinyl ketone (**17**) and cyclopentadiene (**18**) by ^1^H-NMR spectroscopy. Catalysts that caused greater blue shift when treated with the sensor also showed higher activities in the Diels-Alder reaction, corresponding to the more significant LUMO-lowering effect on the ketone. A proportionality between the change in energy of sensor upon binding with the catalyst and the change in the activation energy of the reaction could, therefore, be inferred. Finally, a unified description of reactivity *vs.* sensor measurement was proposed in the form of an equation.

### 2.2. Determination of Catalyst Reactivity: Spectroscopic Methods

**Figure 5:**
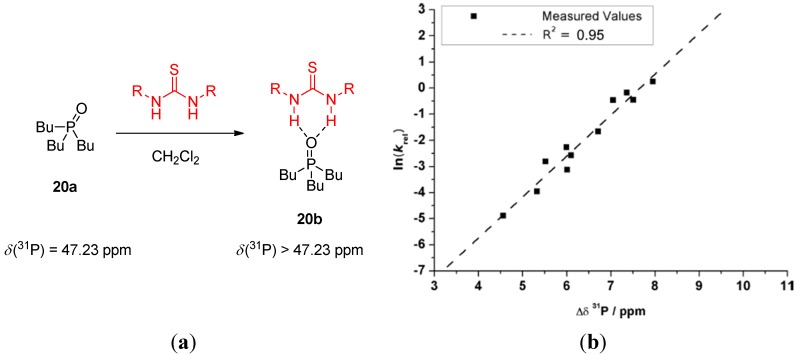
Quantification of H-bond strengths by ^31^P-NMR shifts using **20a** (**a**); the plot of ln(*k*_rel_) values of Diels-Alder reaction of methyl vinyl ketone and cyclopentadiene *vs.* thiourea-induced ^31^P-NMR shifts (**b**). Adapted with permission from Ref. [29]. Copyright (2014) John Wiley and Sons.

Phosphine oxide **20a**, which resembles a carbonyl group, was explored as ^31^P-NMR signal detection-based probe (Figure 5) [29]. Upon formation of the hydrogen bond between a thiourea and the oxygen atom of **20a**, the electron density is shifted towards the thiourea, resulting in the downfield shift of the ^31^P-NMR signal. Again, electron-withdrawing groups attached to the catalysts were found to have a pronounced effect on the NMR signal shift. There is an excellent linear correlation between Δδ ^31^P-NMR and ln(*k*_rel_) of the pseudo-first order Diels-Alder reaction, which confirms the applicability of in this way acquired data. The reaction of methyl vinyl ketone (**17**) and a large excess of cyclopentadiene (**18**) was catalyzed by 10 mol % of a thiourea. Overall, this approach seems more suitable than the method relying solely on the p*K*_a_ values because better correlation was found for all the studied catalyst (*R*^2^ = 0.723 *vs.* 0.307).

**Figure 6:**
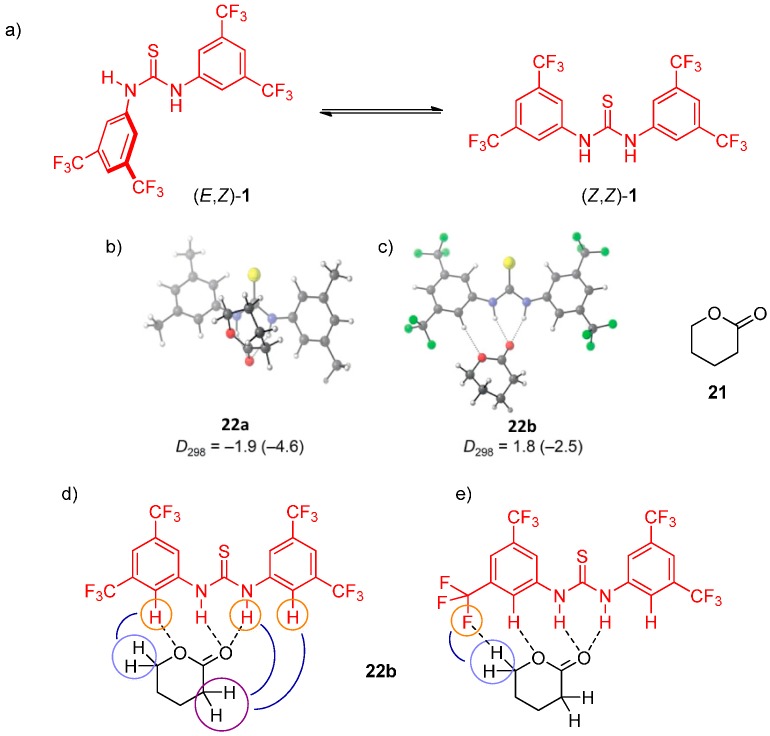
Conformers of thiourea **1**: (**a**) lowest-lying complex **22b**, consisting of thiourea **1** and lactone **21**; (**b**) lowest-lying complex of **1** with lactone **21**; (**c**) important NOESY; (**d**) and ^19^F–^1^H HOESY interactions in complex **22b**; (**e**) Dissociation energies (*D*_298_) are given in kcal·mol^−1^. Values with parentheses were computed with the PCM model for toluene employing UAHF radii. Adapted with permission from Ref. [30]. Copyright (2012) John Wiley and Sons.

The acidity and reactivity enhancing effect of the 3,5-bis(trifluoromethyl)phenyl group on the thiourea catalysts became apparent in all the previous studies. Possible reasons for this include an increase in catalyst polarity, polarizability, acidity, and π-π interactions of the aryl group. Schreiner exploited various spectroscopic and computational techniques to gain deeper insight into its mode of substrate binding [30]. First, it was established that at 298 K thiourea **1** adopts (*Z*,*Z*)-conformation exclusively and transforms into (*E*,*Z*)-conformation only at lower temperatures (below 190 K). A new band for the lactone carbonyl appeared in the IR spectrum upon complexation with lactone **21**, which could adopt two conformations. The stretching vibration appeared at 1712 cm^−1^ for the complexed carbonyl and 1744 cm^−1^ for the uncomplexed lactone. Both signals consist of overlapping peaks for both lactone conformers, providing an indication of a hydrogen-bond complex of lactone **21** and thiourea **1**. Further evidence includes shifts of the ^1^H-NMR signals, most notably that of the catalyst N–H hydrogen (Δδ 1.8 ppm) and the shifts of ^13^C-NMR of carbonyl (Δδ 3.2 ppm) and methylene carbon atom next to the oxygen (Δδ 0.7 ppm). This notion was supported by MS (ESI) measurements, where the complex was identified, and computationally by natural bond order (NBO) analysis. This analysis also confirmed N–H...O=C and even 2.5-fold weaker C–H...O_ring_ interactions [31] (Figure 6), in agreement with quantum theory of atoms in molecules (QTAIM) study. Moreover, NMR techniques such as NOESY and ^19^F–^1^H HOESY supported the proposed complex structure **22b**. Analogous complexes of thiourea **1** with various H-bond acceptors such as *N*-acyl oxazolidinone were similarly observed. However, no such results with lactone **21** were obtained with *N*,*N*′-bis(3,5-dimethylphenyl)thiourea (**23**).

### 2.3. Combined NMR and DFT Approaches in the Mechanism Elucidation

**Figure 7:**
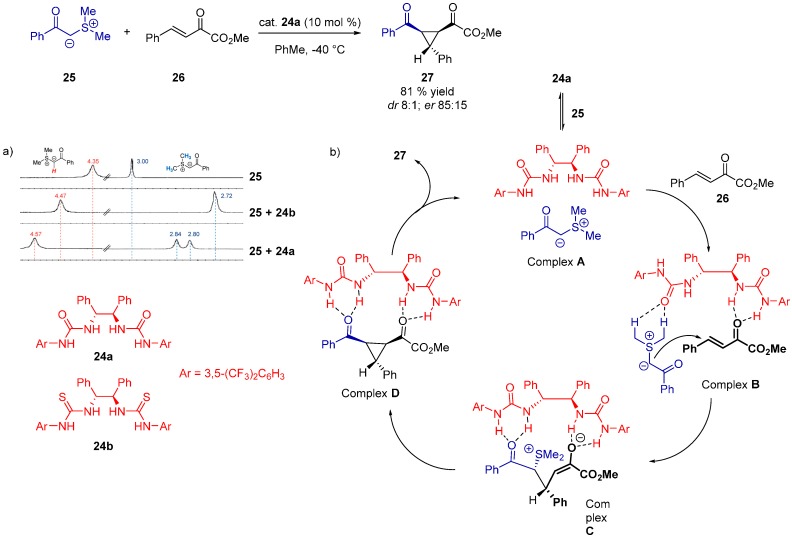
The downfield shift of ^1^H-NMR signal for C–H of sulfur ylide **25** after complex formation with **24a** and **24b** (**a**); proposed catalytic cycle for asymmetric cyclopropanation (**b**). Adapted with permission from Ref. [32]. Copyright (2011) American Chemical Society.

Various experimental and theoretical techniques are often used in conjunction to provide useful results. Such was the case of the asymmetric cyclopropanation of α,β-unsaturated ketoesters with stabilized sulfur ylides, catalyzed by *C*_2_-symmetric ureas [32]. For the ^1^H-NMR studies, thiourea **24b** was used instead of the analogous urea catalyst **24a** due to the solubility reasons. The downfield shift of N–H signals was observed, which indicates that **24b** formed complexes with both reactants and the product. Surprisingly, urea **24a** became soluble in CDCl_3_ when combined with the sulfur ylide **25**, resulting in a remarkable shift of the ylide C–H. Based on these findings and the DFT calculations (B3LYP functional, structures optimized in gas phase with the 6-31G(d,p) basis set), a catalytic cycle was proposed (Figure 7).

**Figure 8:**
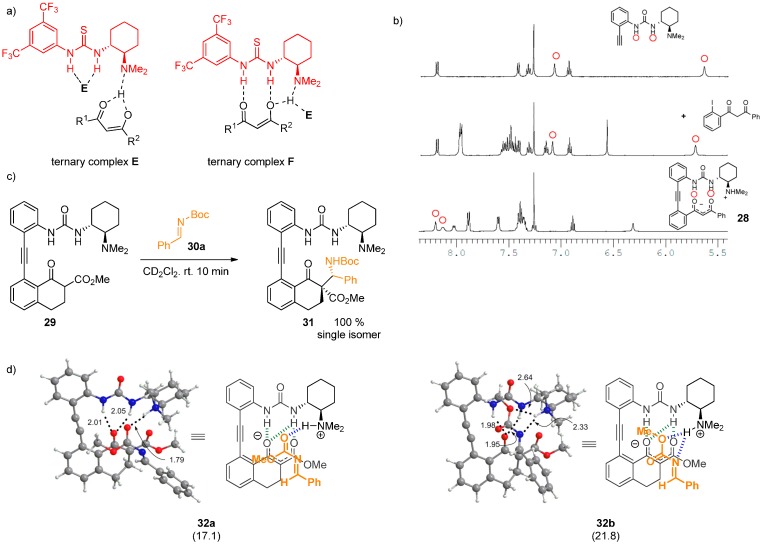
Ternary complexes of thiourea with 1,3-dicarbonyl compound (**a**); ^1^H-NMR shifts in **28** (**b**); reaction of **29** with an electrophile (**c**); two proposed channels of imine coordination (**d**). Adapted with permission from Ref. [19]. Copyright (2014) American Chemical Society.

Takemoto and coworkers investigated the bifunctional nature of the catalysts [19]. Several binary catalysts were designed, in which a urea moiety was linked to a 1,3-dicarbonyl compounds. These could mimic the intermediates formed during the reaction of a thiourea and 1,3-dicarbonyl compound. The thermodynamic stability of such compounds facilitates their spectroscopic studies, which could hence distinguish between the formation of ternary complexes **E** and **F**. First, ^1^H-NMR N–H signal downfield shifts were observed for different compounds containing 1,3-dicarbonyl unit such as 1,3-diketones or β-ketoesters compared to separate unit-containing compounds, which confirmed the presence of an H-bond (Figure 8). The X-ray structure of **28** demonstrated the complete formation of an ammonium enolate by deprotonation of the 1,3-dicarbonyl with the dimethylamino group and double H-bonding to both oxygen atoms from ammonium N–H and one of the urea’s N–H. Analogous β-ketoester derivative **29** afforded products as single isomers upon reaction with various electrophiles, e.g., **30a**. Next, a reaction mechanism for the Mannich reaction of **29** with *N*-methoxycarbonyl imine **30b** was studied by DFT methods (B3LYP/6-31G*). Two channels **31a** and **31b** of proton coordination of two different imine configurations were suggested. The overall results were in accordance with the formation ternary complex **F**, which had been already proposed by Pápai [33].

Smith has found that the catalyst efficiency could be enhanced substantially by cooperative effects and designed a new type of monofunctional catalysts, where a thiourea moiety is activated by H-bonding from a linked urea moiety [34]. These catalysts could mimic protein folding and thus have the following potential advantages: (1) preorganization of the catalyst by direct folding, stabilized by H-bonds, thus reducing the entropic cost of transition state binding; (2) formation of stronger intramolecular noncovalent interactions, leading to cooperative ligand binding and greater stabilization of the transition state. NMR studies of compound **33** revealed the presence of multiple intramolecular H-bonds, with the catalyst adopting (*E*,*E*)-conformation of the thiourea. Very high levels of activity and selectivity were achieved with low catalyst loading (1 mol %) in the Mannich reaction between aromatic aldimines **30a** and silyl ketene acetal **34**.

**Figure 9:**
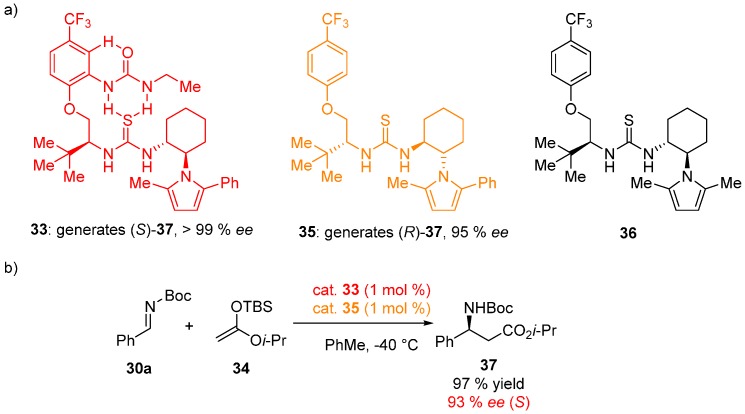
Structures of the catalysts used in the study (**a**); Mannich reaction kinetic experiment demonstrating higher activity of catalyst **33** (**b**).

Kinetic experiments were conducted to probe the catalyst efficiency. A competition experiment with catalysts **33** (1 mol %, gives (*S*)-product) and **35** (1 mol %, gives (*R*)-product) furnished (*S*)-product in 97% yield and 93% *ee*, which signifies that catalyst **33** outperforms derivative **35** in this transformation (Figure 9). This fact is likely the result of stronger noncovalent interactions present in catalyst **33**, which are absent in compound **35**, expressed in the term of tighter ligand-acceptor binding and rate enhancement. Furthermore, the anion-binding ability was tested by observing the complex formation with chloride anion by NMR. A 1:1 complex formation with chloride anion with *K*_a_ = (550 ± 85) M^−1^ was measured for catalyst **35**, consistent with the formation of two N–H…Cl bonds. In contrast, compound **36**, without cooperative interactions, has a *K*_a_ < 10 M^−1^. An NMR titration experiment in CDCl_3_ with DMSO-*d*_6_ as an H-bond acceptor, NOE correlations, and IR spectra confirmed the presence of internally bonded urea hydrogens and the thiourea hydrogens available for the guest.

**Figure 10:**
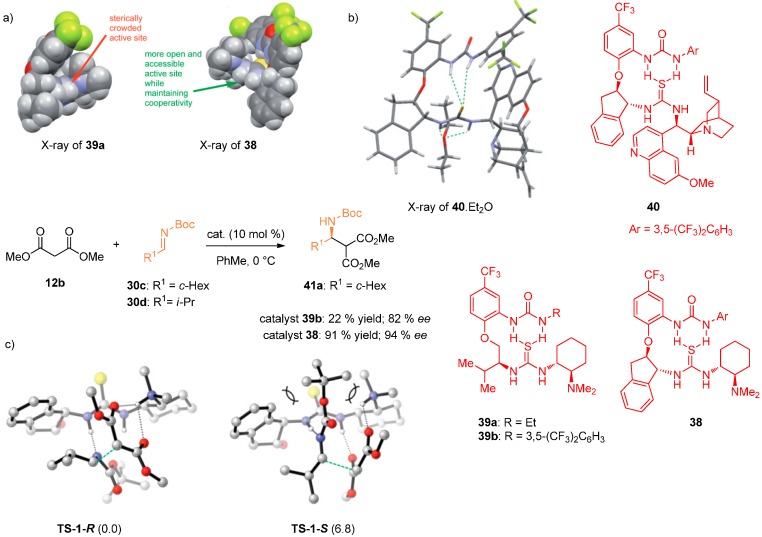
Catalyst design and more accessible thiourea moiety in **38** compared to **39a** (**a**); X-ray of complex **40**.Et_2_O (**b**); DFT-calculated transition states for the Mannich reaction between imine **30d** and dimethyl malonate (**12b**) (**c**). The urea moiety of the catalyst is omitted for clarity. Computed relative energies are shown in parentheses (kcal·mol^−1^). Adapted with permission from Ref. [35]. Copyright (2012) John Wiley and Sons.

Pihko and coworkers further explored this type of catalysts. They developed bifunctional catalysts based on the same design [35]. The X-ray structure of **38** with more rigid *trans*-1,2-aminoindanol linker showed, compared to catalyst **39a** with amino alcohol linker, that the thiourea moiety is more exposed for simultaneous activation of both substrates. This can also be seen in X-ray structure of catalyst **40** with diethyl ether. Catalyst **38** also exhibited high activity in the Mannich reaction between aliphatic aldimines and malonates. The superior performance of catalyst **38** over catalysts **39** was shown by monitoring the reaction between aliphatic aldimine **30c** and malonate **12b** by NMR spectroscopy (Figure 10). DFT calculations (geometry optimization at the B97D/6-31G * level; single-point energies at M06-2X/6-311++G **; solvent effects of toluene were considered using IEFPCM method) were carried out to investigate the mode of substrate activation. Imine nitrogen can be coordinated by only one H-bond, requiring the displacement of the malonate from its optimal position, already coordinated by two H-bonds. However, even this single H-bond to the imine can significantly activate the substrate if another intramolecular H-bonding enhances the H-bond donor capacity of the thiourea. The calculations demonstrated that **TS-1-*R*** is preferred over **TS-1-*S*** due to unfavorable steric interactions present in **TS-1-*S***.

**Figure 11:**
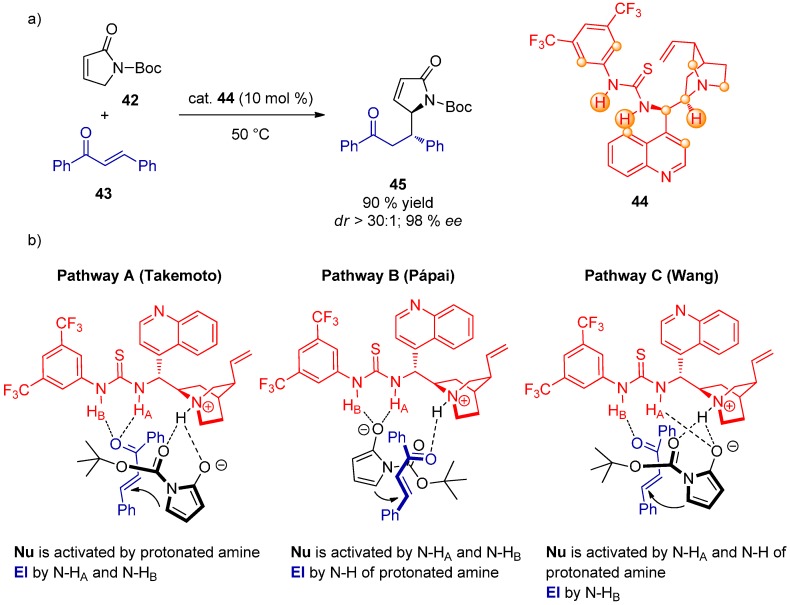
Dual activation mechanism TS structures of the C–C bond forming step for the addition of γ-butyrolactam **42** to chalcone **43** (**a**); proposed mechanism pathways (**b**).

A dual activation mechanism for a Michael addition of γ-butyrolactam **42** to chalcone **43** was explored in a detailed study exploiting NMR and DFT calculations (B3LYP/6-31G(d,p) using CPCM model with CHCl_3_ as solvent; energies from B3LYP/6-311++G(d,p) (CPCM, CHCl_3_)//B3LYP/6-31G(d,p) (CPCM, CHCl_3_) [36]. The authors proposed a new activation pathway and presented the evidence for the simultaneous activation of both the nucleophile and the electrophile by the thiourea moiety. Stronger interactions of catalyst **44** with the nucleophile **42**, in comparison to the interactions with the electrophile **43**, were discovered by ^1^H-NMR (downfield-shifted protons are marked in orange in the structure of **44**) and confirmed by DFT methods (Figure 11). Variable-temperature NMR kinetic studies revealed that the reaction is first-order in both the nucleophile and the electrophile, as well as the catalyst, meaning the C–C bond forming step is probably rate-determining. Three possible pathways **A**–**C** of substrate activation were then assessed. Pathway **A** was found to converge to path **C** by DFT calculations while pathways **B** and **C** resulted in the primary product. The main product was predominantly formed by pathway **C**, that is by the simultaneous activation of the nucleophile by N–H_A_ of the thiourea and N–H of the protonated amine of the catalyst. The other N–H_B_ bond of the thiourea activates the electrophile. This notion is supported by the fact that N–H_B_ is more acidic than N–H_A_ and thus might act differently. C–H...O interaction in pathway **C** was also discovered. The diastereoselectivity of the reaction was explained by unfavorable steric interactions present in the TS leading to the other diastereomer. The calculated stereoselectivities (100% *ee*, >60:1 *dr*) matched the experimental data (98% *ee*, >30:1 *dr*).

**Figure 12:**
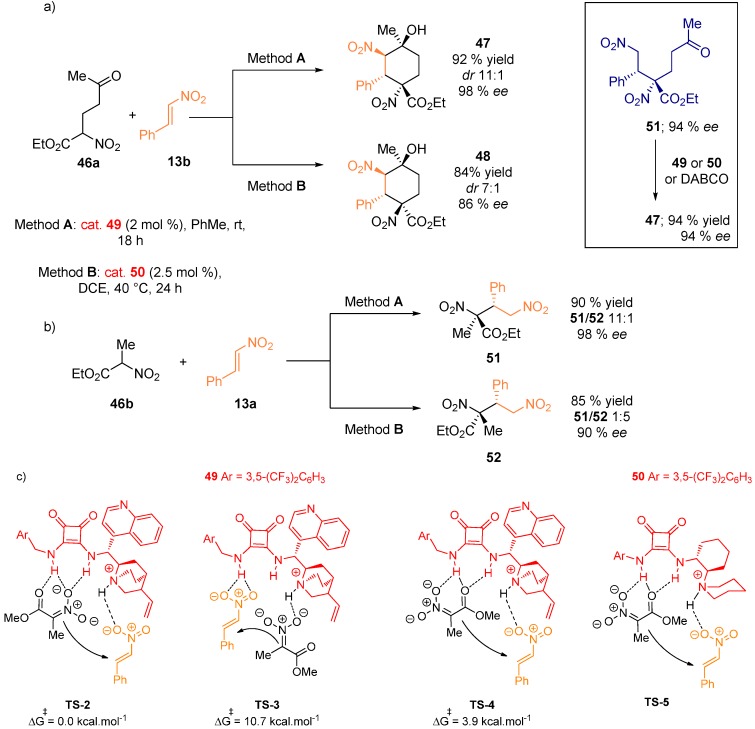
Diastereodivergent Michael addition for the synthesis of substituted cyclohexanes (**a**); synthesis of simplified products (**b**); transition states with catalysts **49** and **50** (**c**).

The diastereodivergent process leading to densely functionalized cyclohexanes from δ-oxo-α-nitrohexanoate **46a** and nitrostyrene **13b** was reported (Figure 12) [37]. Two different diastereomers **47** and **48** were isolated in high enantiomeric excesses, depending on the use of the bifunctional squaramide catalysts **49** or **50**. Kinetic experiments suggested the Michael addition is the rate-determining step. To explain the observed different diastereoselectivity, intermediate **51** was prepared and treated with either **49**, **50** or DABCO. The reaction furnished the same diastereomer, indicating that after the formation of two stereocenters governed by the catalyst, the substrate controls the diastereoselectivity. ^1^H-NMR experiments displayed the squaramide-nitronate complex to be formed while the corresponding complex formation from the nitrostyrene **13b** was not observed. DFT studies (B3LYP/6-31G(d) using PCM model) to explain these results were then conducted on simplified substrate **46b**. Two possible pathways were evaluated for the reaction catalyzed by squaramides **49**. The nitroalkene can be activated by the squaramide and the pronucleophile by the interaction with the amine functionality of the catalyst (**TS-3**). Alternatively, the deprotonated nucleophile can be coordinated with the squaramide moiety and the nitroalkene can engage in the H-bonding interaction with the ammonium group (**TS-2**). Calculations showed that **TS-2** is favored by more than 10 kcal·mol^−1^ (41.8 kJ·mol^−1^). Transition state **TS-4** leading to the other diastereomer is higher in energy by 3.9 kcal·mol^−1^ (16.3 kJ·mol^−1^), which is in agreement with the experimental results. The calculations were repeated with the catalyst **50**, again resulting in a similar activation of the nucleophile (**TS-5**).

### 2.4. Combined Spectroscopic and Kinetic Studies Investigations

**Figure 13:**
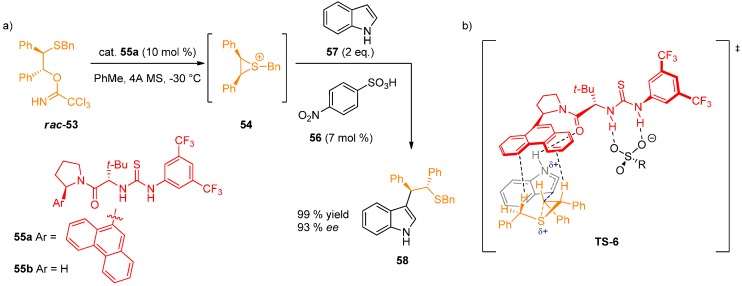
Thiourea-catalyzed ring opening of episulfonium ions (**a**); proposed transition structure model **TS-6**, stabilized by attractive, non-covalent interactions (**b**).

Jacobsen reported a thiourea-catalyzed ring opening of episulfonium ions with indoles [38]. Specifically, racemic trichloroacetamidates **53** form episulfonium ions **54** under the catalysis of an aryl pyrrolidine-derived thiourea **55a** and 4-nitrobenzenesulfonic acid (**56**) (Figure 13). Intermediates **54** then undergo nucleophilic ring opening with an indole derivative **57** to give products in excellent yields and enantioselectivities. In the comprehensive study, several stabilizing non-covalent interactions were analyzed [39].

First, the enantioselectivity- and rate-determining step was identified by reaction progress kinetic analysis using *in situ* infrared spectroscopy. First-order rate dependence on sulfonic acid **56**, catalyst **55a** and indole was discovered. The reaction of precursor **53** and acid **56** rapidly afford compound **54**. These results indicate that the episulfonium **54** is the resting state of the substrate and that indole is involved in the rate-determining transition structure. Linear correlations were observed between the reaction rate and Mayr’s nucleophilicity parameter *N* for 5 different indole derivatives for both racemic and thiourea**-**catalyzed reaction. Kinetic isotopic effect measurements with 3-deuterioindole showed almost negligible effect of the isotopic substitution, therefore excluding the re-aromatization as the rate-determining step, leaving only the indole addition step for further consideration. In the next step, the following catalyst-substrate interactions were investigated:

(1) Thiourea double H-bond interaction with the sulfonate anion: Determined by ^1^H-NMR titration of catalyst **55a** with (Bn_2_SMe)^+^(OTf)^−^ (**59**), leading to the formation of 1:1 complex accompanied by sharpening and downfield shift of N–H protons. This is supported by the observed dependence of *ee* on the sulfonate anion structure.
(2) H-bond interaction with the indole N–H: In contrast to indole, *N*-methylindole and the series of π-nucleophiles without N–H group in a 1,3-relationship gave very low enantioselectivities. The acceleration rate for the asymmetric reaction log(*k*_asym_/*k*_rac_) was found to be linked linearly with the p*K*_a_ of 5-substituted indoles. This is in accordance with a general base activation of indole by the catalyst. After ruling out several possibilities experimentally, the authors proposed that the catalyst amide oxygen-indole N–H H-bond is responsible for this activation.
(3) Stabilization of the cationic transition state by cation-π interactions: Strong correlation was observed between the enantioselectivity of the reaction and the arene unit of the catalyst. The pathway leading to minor enantiomer also shows a positive correlation, indicating stabilization of the minor transition structure, although to a lesser degree. ^1^H-NMR study of 1:1 complex formation between **59** and thiourea derivatives **55a** and **55b** was conducted. Thiourea **55a** caused a upfield shift of the benzylic and methyl protons of **59** by 0.6–0.8 ppm, whereas **55b** without aryl substituents had no effect on the chemical shift of the protons. These data suggest that attractive π-interactions between the arene and the sulfonium ion are responsible for the observed enantioselectivity, with more extended aromatic substituents resulting in an increase in cation-π interactions.

After taking all this experimental evidence into consideration, the authors proposed a transition-structure model **TS-6** (Figure 13).

Cation-π interactions of aryl pyrrolidine thiourea catalysts were also assessed in enantioselective cyclization of hydroxylactams [40]. Eyring analysis revealed an enthalpic effect of the arene unit, thus pointing to the energetic stabilization of cationic intermediates. Subsequently, a linear correlation was noted in the plot of enantioselectivity (expressed in ΔΔG**^†^**) as a function of arene polarizability. Such parameters are seldom used to invoke correlations to catalyst performance [41].

**Figure 14:**
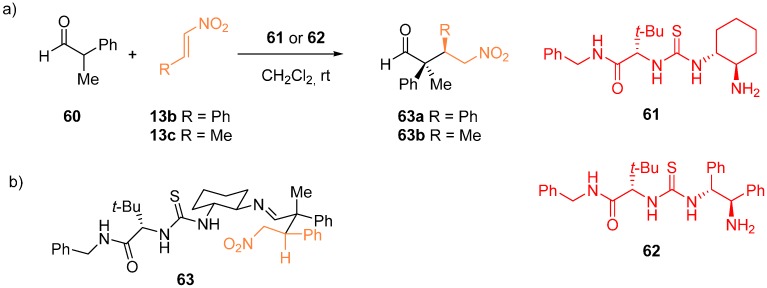
Enantioselective conjugate addition of α,α-disubstituted aldehydes to nitro-olefins (**a**); detected iminium intermediate (**b**).

Reversibility was reported to be playing a significant role in the enantioselective conjugate addition of α,α-disubstituted aldehyde **60** to nitro-olefins **13** catalyzed by primary amine thioureas (Figure 14) [42]. Both catalysts **61** and **62** gave product **63a** starting from **13b** in excellent diastereo- and enantioselectivity, however, only catalyst **62** was active when substrate **13c** was employed. Intermediate **63**, formed by the reaction between **60** and **13b**, identified by the combination of 2D NMR experiments, is supposed to be the resting state. By conducting various kinetic experiment studies including the reverse reactions with both racemic and enantiopure products and the catalysts, the authors observed e.g., strong kinetic resolution in the reaction of catalyst **62** and racemic product **63b** with *k*_rel_ > 25 and only slight erosion of *ee* in the reaction of the catalyst **62** with enantiopure **63b**. More significant erosion of *ee* was detected with catalyst **61**. Two levels of stereoselection were established: (1) the enamine formation from the imine (either *E*- or *Z*-); and (2) the addition of the nitro-olefin, creating the second chiral center. The observed differences in stereoselectivity were ascribed to the degree of reversibility in the specific catalyst/substrate combinations: In some combinations all reaction steps are reversible, whereas in others only the iminium hydrolysis displays reversibility.

### 2.5. Computational and Kinetic Studies

**Figure 15:**
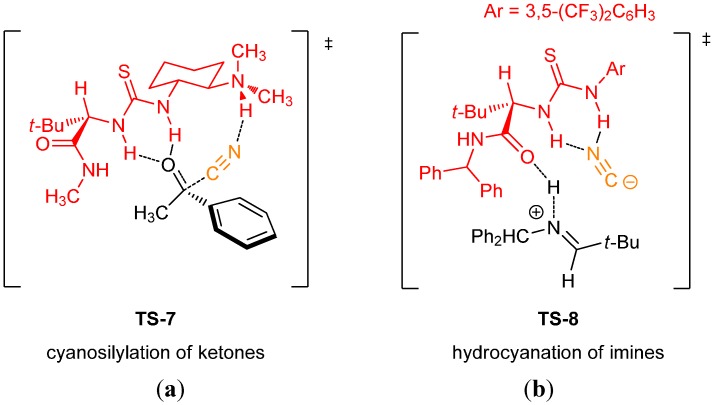
Transition states for the cyanosilylation of ketones (**a**); and hydrocyanation of imines (**b**).

The mechanisms of cyanosilylation of ketones and hydrocyanation of imines were studied extensively using experimental kinetic analysis and DFT methods (B3LYP/6-31G(d)) [43,44]. The transition states proposed for both reactions are depicted in Figure 15. The mechanism of the former proceeds by **TS-7**, binding the ketone first by the thiourea hydrogens. In contrast, the latter proceeds preferentially by **TS-8**, binding of the cyanide ion by the thiourea, indicating that the thiourea acts as an anion receptor and not as the electrophile activating group. Based on this studies, catalysts with more sterically demanding amides were designed for the cyanosilylation of more challenging dialkyl ketones.

### 2.6. Combined Spectroscopic, Kinetic and Computational Studies

**Figure 16:**
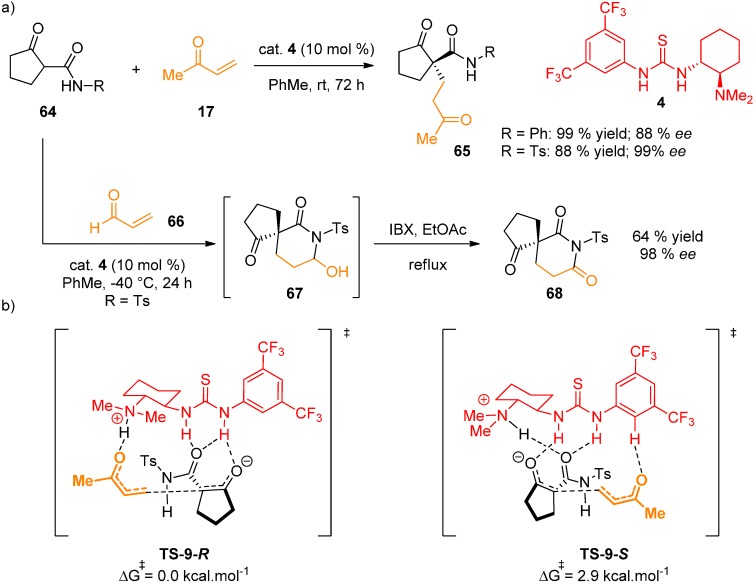
Michael addition of β-ketoamides **64** to α,β-unsaturated carbonyl compound **17**, catalyzed by **4** (**a**); calculated transition states (**b**).

Michael addition of β-ketoamides **64** to α,β-unsaturated carbonyl compound **17** catalyzed by Takemoto thiourea **4** was reported (Figure 16) [45]. First, to determine the absolute configuration of the products, the authors utilized a combination of X-ray and VCD spectroscopy. The following ^1^H-NMR studies confirmed the presence of multiple H-bonds and possible aryl π-π interactions between the substrate **64** and the catalyst **4**. Kinetic experiments showed positive rate-order for **17** and **4**, but zero rate order for **64**. DFT calculations (M06-2X/6-31G(d) basis set; full geometry optimization in toluene using IEFPCM model) were then used to establish the reaction mechanism. The tautomeric equilibrium and the deprotonation of **64**, C–C bond formation and the reprotonation of the product were assessed. For the C–C bond formation step, two transition states **TS-9-*R*** and **TS-9-*S*** were proposed (Figure 14), with a calculated ΔΔG**^†^** difference of 2.9 kcal·mol^−1^ (12.1 kJ·mol^−1^), in accordance with the observed enantioselectivity (R = Ts; 99% *ee*). A network of H-bonds is responsible for the stabilization of the transition state in which **64** has a perpendicular orientation with respect to **4**, with additional stabilization provided by π-π interactions of the aryl rings. Reprotonation occurs by abstraction of the amide proton by the enolate first, followed by proton transfer from the ammonium to the amide, justifying the need for relatively acidic amide N–H proton. Thermodynamically demanding liberation of the catalyst from the amide was identified to be the limitation of the reaction. However, a strategy was devised relying on the formation of cyclic hemiaminal by the intramolecular addition of the amide to the carbonyl, hence shifting the equilibrium towards the catalyst release. This idea was put into practice and indeed, higher reaction rates were observed for the reaction of **64** and aldehyde **66**, forming hemiaminal structures **67**.

## 3. Computational Methods

The rapid rise of computational power enabled the spread of advanced computational protocols for a range of chemistry problems. Large assemblies of chiral organocatalysts together with functionalized substrates can be investigated almost routinely by DFT calculations [46,47,48]. Noncovalent organocatalysis using H-bonding was reviewed only once, but its focus was rather narrow [49].

**Figure 17:**
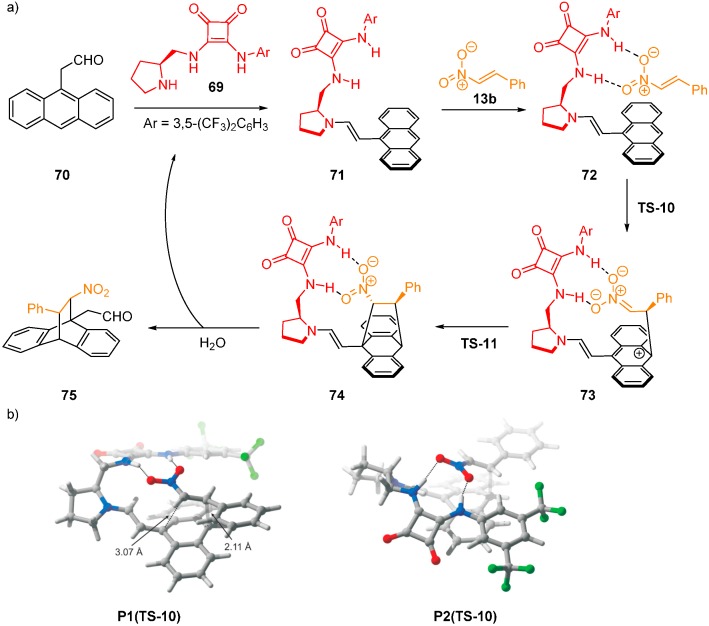
Diels-Alder reaction of anthracene **70** and nitroalkene **13b** (**a**); optimized structures of **P1(TS-10)** and **P2(TS-10)** (**b**). Adapted with permission from Ref. [50]. Copyright (2013) John Wiley and Sons.

A comparison of (thio)urea and (thio)squaramide performance with squaramide **69**, used in a Diels-Alder reaction between anthracene **70** and nitroalkene **13b**, was reported (Figure 17) [50]. During the reaction, the enamine activation via HOMO raising of **70** occurs in the form of **71**, and intermediate **73** is formed via **TS-10**. The stereochemistry is controlled by H-bonding between the squaramide and nitro group of the dienophile. This coordination is followed by the formation of the other bond via **TS-11** to **74** and subsequent hydrolysis of the enamine to regenerate the catalyst. **TS-10** is higher in energy and, therefore, rate-limiting for both pathways. **P1(TS-10)** leading to the major enantiomer is 4.2 kcal·mol^−1^ (17.6 kJ·mol^−1^) lower in energy than **P2(TS-10)** leading to the minor enantiomer ((B97-D/TZV(2d,2p); single point energies at ω-B97X-D/TZV(2d,2p); solvent (dichloromethane) was accounted for using the PCM method). A distortion/interaction analysis rationalized the reason behind the difference. In this approach, the gas-phase energy barrier is split into the distortion energy (required to distort each reactant into TS geometry) and the energy gained through the interactions of the distorted fragments. The interaction energies for compounds **75** (**P1**) and *ent*-**75** (**P2**) were found to be similar. However, the distortion energies differ by 3.6 kcal·mol^−1^ (15.1 kJ·mol^−1^). The extra distortion energy present in **P2(TS-10)** is caused by an unfavorable conformation of the squaramide linkage in order to maintain the H-bonding to the dienophile. In addition, anthracene **70** does not react with methyl styryl sulfone under these conditions. This lack of reactivity is caused by the higher energy required by the sulfone to distort to TS geometry, leading to significant deformation and disruption of conjugation.

**Figure 18:**
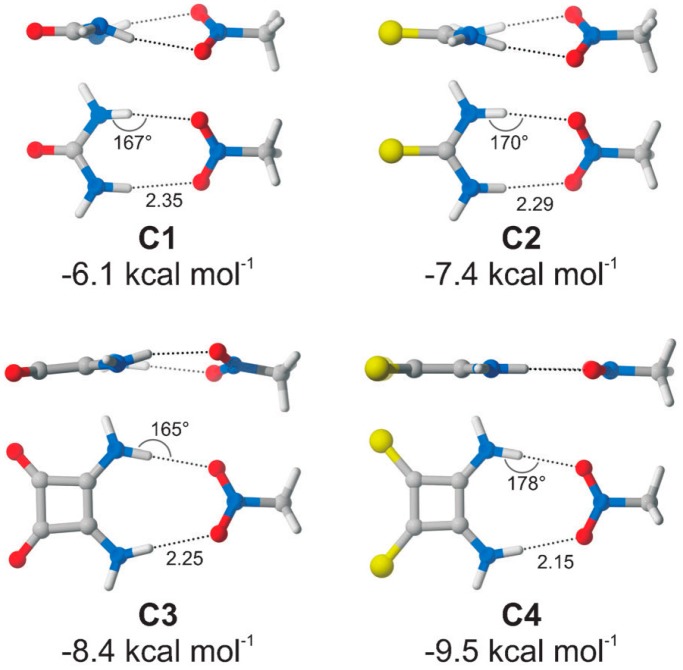
Four complexes **C1**–**C4** of (thio)ureas and (thio)squaramides with nitromethane. Adapted with permission from Ref. [50]. Copyright (2013) John Wiley and Sons.

Four complexes of thioureas and thiosquaramides **C1**–**C4** with nitromethane were then evaluated (Figure 18). The calculated binding energies decrease in the order **C4** > **C3** > **C2** > **C1**. Complex **C4** has the shortest H-bond lengths and almost linearly aligned N–H...O atoms. The replacement of oxygen by sulfur leads to bond strength enhancement by 1 kcal·mol^−1^ (4.2 kJ·mol^−1^) (smaller difference between p*K*a of an H-bond donor and an acceptor leads to stronger H-bond), while the interactions with thiosquaramides are by >2 kcal·mol^−1^ (8.2 kJ·mol^−1^) more favorable over the corresponding thioureas. Based on these results, the authors predicted that thiosquaramide-catalyzed reaction between **70** and **13a** should have lower energy barrier than the squaramide **69**-catalyzed reaction by 2.5 kcal·mol^−1^ (10.5 kJ·mol^−1^), which in turn has lower energy barrier than thiourea-catalyzed process.

**Figure 19:**
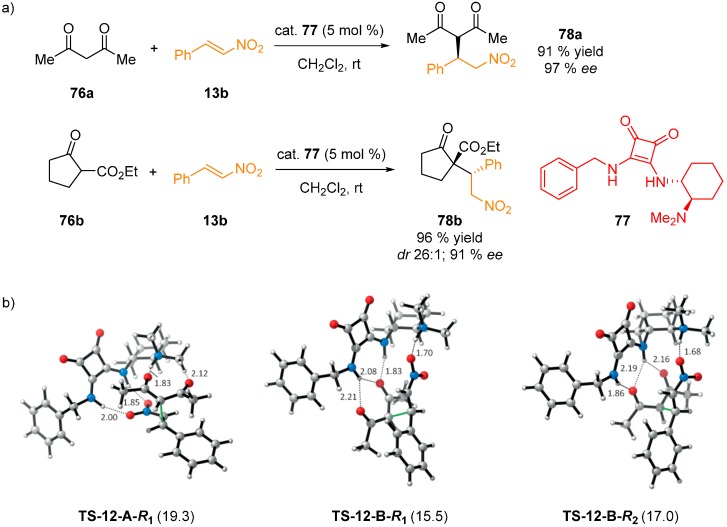
Michael reaction of acetylacetone (**76a**) and **76b** to nitrostyrene (**13b**) (**a**); selected calculated transition states for pathways **A** and **B** leading to **78a** (**b**). Adapted with permission from Ref. [51]. Copyright (2014) John Wiley and Sons.

Three possible substrate activation pathways (see above) were challenged in the Michael addition of 1,3-dicarbonyl compounds **76** to nitrostyrene **13b**, catalyzed by chiral squaramide **77** (Figure 19) [51]. First, the conformational analysis of the catalyst was conducted and then each pathway was analyzed (B3LYP/6-31G(d); single-point energies at M06-2X/6-311++G(d,p); solvent effects (CH_2_Cl_2_) using IEFPCM). For the addition of acetylacetone (**76a**) to nitrostyrene **13b**, both reactants were able to form double H-bond with the squaramide. Pathways **A** and **B** were located, however, pathway **C**, as proposed by Wang, could not be identified. Four transition state structures were proposed for each pathway, with **TS-12-B-R_1_** being the lowest in energy and responsible for 92% of the Boltzmann distribution. Transition state **TS-12-A-R_1_** is 3.8 kcal·mol^−1^ (15.9 kJ·mol^−1^) less stable. Another transition state **TS-12-B-R_2_** was only 1.5 kcal·mol^−1^ less stable. The bifunctional squaramides, therefore, provide multiple reaction channels leading to the same product. The same procedure was repeated with ethyl 2-oxocyclopentanecarboxylate (**76b**) as the nucleophile, explaining the observed enantio- and diastereoselectivity. In summary, a structurally rigid chiral oxyanion hole, likewise in some enzymes, was suggested by overlay analysis of the feasible transition states. Pathway **B**, the activation of the electrophile by the protonated amine, was found to be preferred. However, various reactivity models should be evaluated in each case, as squaramides offer increased H...H distance in comparison to thioureas, thus making alternative binding modes accessible.

**Figure 20:**
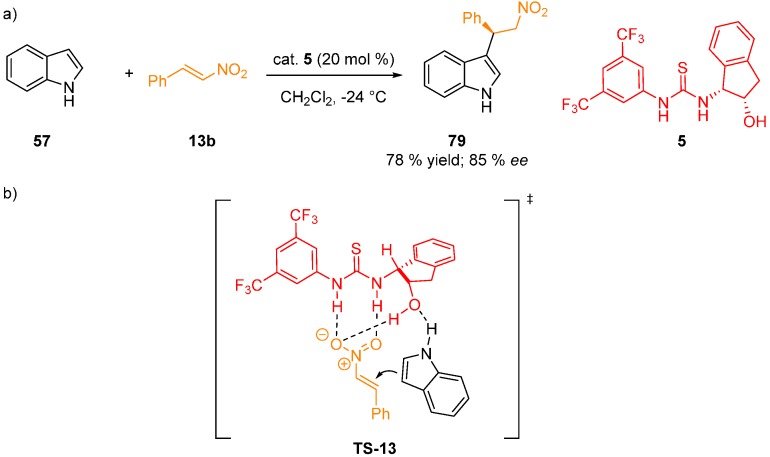
Friedel-Crafts alkylation of indole (**57**) (**a**); calculated transition state (**b**).

A theoretical study of the Friedel-Crafts alkylation of indole (**57**) with nitrostyrene (**13b**) using *cis*-aminoindanol-derived catalyst **5** was reported (Figure 20) [52]. DFT calculations (M06-2X/6-311G(d,p) level; solvation using PCM(CH_2_Cl_2_)) revealed that in the most stable transitions state **TS-13**, indole **57** coordinates to the hydroxyl group of the catalyst and the nitroalkene is coordinated perpendicularly via double H-bonds to the thiourea. Additional activating H-bond between the hydroxyl group and the oxygen atom of the nitroalkene was discovered. It explains the absence of reactivity if O–H functionality is not present on the catalyst (TS for the uncatalyzed reaction is 17 kcal·mol^−1^ (71.1 kJ·mol^−1^) higher in energy) or is in the *trans-*configuration with respect to the thiourea moiety. The calculations also explained higher reaction rate and lower selectivity of this process when 2-methylindole was used as the nucleophile.

**Figure 21:**
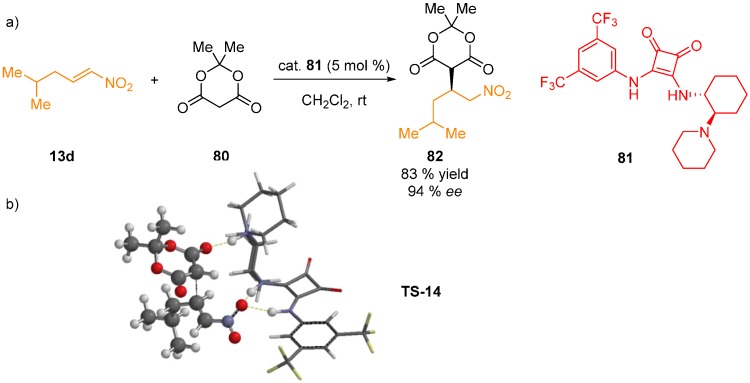
Michael addition of Meldrum’s acid **80** to nitroalkene **13e** (**a**); proposed transition state model (**b**).

In our group, a theoretical investigation of the squaramide-catalyzed Michael addition of Meldrum’s acid **80** to nitroalkene **13d** in the synthesis of pregabalin by DFT method was conducted (BP86/def2-SVP) [53]. In the proposed transition state model, nitroalkene forms two H-bonds to the squaramide moiety, while the anion of Meldrum’s acid is bound via one of the carbonyl oxygen atoms through H-bond to the protonated piperidine unit of the catalyst (Figure 21). The calculations also showed weak C–H…O interaction between the nitroalkene oxygen atom and 3,5-bis(trifluoromethyl)phenyl group of the catalyst.

**Figure 22:**
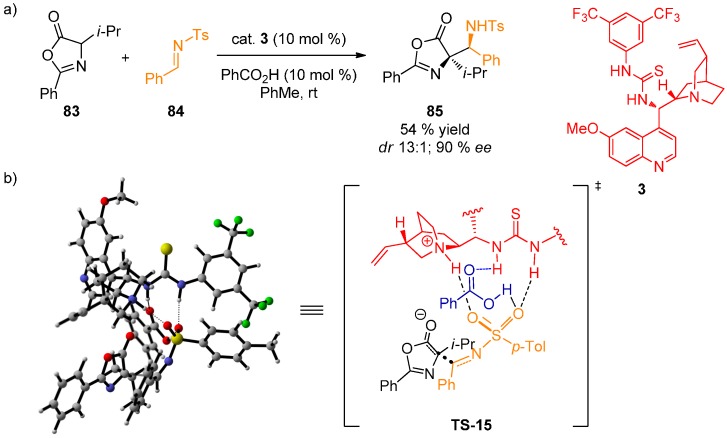
Mannich reaction of oxazolone **83** and *N*-protected imine **84** (**a**); proposed transition state model (**b**).

A Mannich reaction between oxazolones **83** and *N*-protected imines **84**, catalyzed by thiourea catalyst **3**, was reported by our group (Figure 22) [54]. It was discovered that the reaction proceeded with high stereoselectivity only if benzoic acid as a co-catalyst was employed. Interestingly, only racemic product **85** was obtained in the absence of the acid co-catalyst. This was rationalized by a transition state model **TS-15** based on Hartree-Fock calculations using 3-21G basis set. Sulfonyl imine **84** is coordinated to the thiourea moiety as well as the quinuclidine ammonium group via sulfonyl oxygen atoms while benzoic acid activates the imine by additional H-bond. Steric factors govern the approach of the oxazolone.

Computational approaches are now often employed in determining reaction mechanisms and explaining the stereochemical outcome in H-bonding organocatalytic reactions. Quantum-chemical calculation were used for the study of the Henry reaction catalyzed by cyclophane-based bis(thioureas) [55], kinetic resolution of amines [56], dual H-bond/enamine catalyzed domino reaction [57], hydrophosphonylation of a ketoester [58], Michael reactions of α′-hydroxy enones [59], formal [2 + 2] cycloadditions [60], thiopyrano-indole construction process [61], and Mannich-type reaction catalyzed by primary amine thiourea [62].

## 4. Conclusions

Despite inherent difficulties with mechanistic studies of hydrogen-bonding organocatalysis, quite a large array of studies has been undertaken to elucidate mechanistic details. Spectroscopic methods helped to determine acidities of hydrogen-donating organocatalysts. NMR and IR studies were used for determination of possible interactions between catalysts and substrates. Computational methods help in the elucidation of features not available by experimental techniques. Quantum chemical calculations can now also be used for stereochemistry studies. On the other hand, there are still large areas of hydrogen-bonding organocatalysis only poorly understood. Precise bonding of catalysts with substrates is still elusive. In addition, stereochemical models, even supported by computational studies are often only speculative. We believe that this feature article will help the organocatalytic community in further studies of hydrogen bonding organocatalysis. Detailed knowledge of reaction mechanisms, arguably, shall enable further development of this exciting field of research.
